# Papillary Thyroid Cancer in a Patient With Graves’ Disease and Hyperfunctioning (Hot) Thyroid Nodules: An Unexpected Presentation

**DOI:** 10.7759/cureus.64373

**Published:** 2024-07-11

**Authors:** Fares Jamal, Narek Hakobyan, Samrah Siddiqui

**Affiliations:** 1 Internal Medicine, Brookdale University Hospital Medical Center, Brooklyn, USA

**Keywords:** thyroid nodule, thyroid cancer, ultrasonography, graves' disease, thyroid-stimulating immunoglobulin (tsi), thyroid-stimulating hormone, hot nodules, papillary carcinoma of thyroid

## Abstract

Hyperfunctioning (hot) nodules are considered benign while cold nodules are associated with a higher risk of thyroid cancer. In this case report, we present a patient diagnosed with Graves’ disease and later found to have papillary thyroid carcinoma (Bethesda VI), confirmed by fine needle aspiration (FNA) biopsy, with regional metastasis to the neck and possible metastasis to the lungs. This paper demonstrates that hot nodules are not always benign, and could be associated with malignancy.

## Introduction

Thyroid nodules are very common, affecting up to 68% of the population [1]. Hyperfunctioning (hot) nodules have been historically thought to be benign [2]. Conversely, cold nodules and elevated thyroid-stimulating hormone (TSH) are associated with an increased risk of thyroid cancer [2]. However, there is growing recognition that hyperfunctioning nodules and malignancy are not mutually exclusive, and this case report represents another such instance [3]. 

## Case presentation

**Figure 1:**
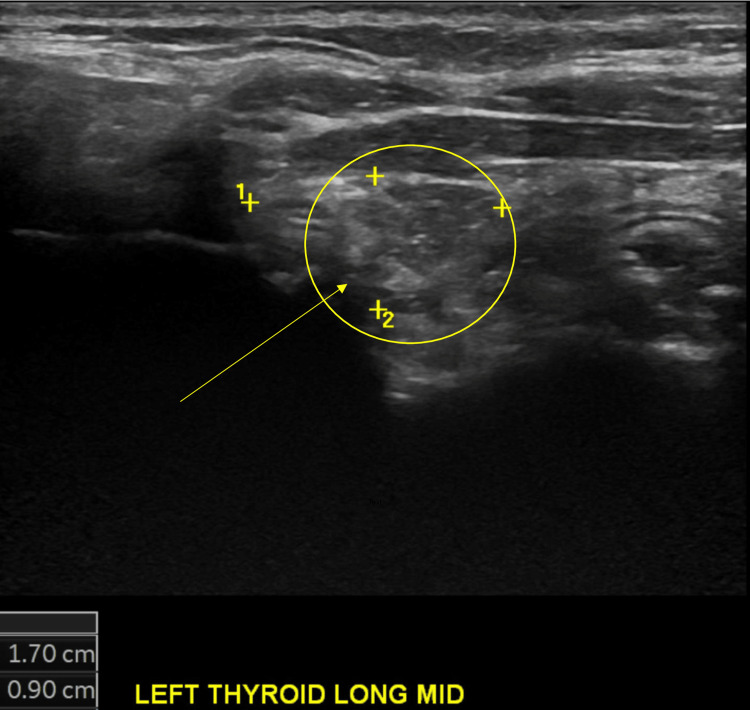
Ultrasound of the left thyroid. The circle contains multiple left thyroid nodules. The arrow is pointing to the largest left thyroid nodule.

A 67-year-old female with a past medical history of coronary artery disease (CAD), chronic kidney disease (CKD) Stage IV, chronic obstructive pulmonary disease (COPD), hypertension, and fibromyalgia, presented with palpitations and unintentional weight loss of 30 lbs over one year. Labs showed suppressed TSH (<0.015 mIU/L) and elevated free thyroxine (FT4) (2.5 ng/dL). Graves’ disease was diagnosed with elevated thyroid-stimulating immunoglobulins (TSIs) at 369% and thyroid peroxidase (TPO) antibodies at 567 IU/mL (Table 1). A thyroid ultrasound (US) showed an enlarged left thyroid containing multiple nodules, with the largest > 2 cm and hypoechoic (Figure 1). A thyroid uptake scan showed increased uptake (46%; normal: 8-35%) concentrated in the upper and mid-left lobes where the nodules were located. Our patient was treated with methimazole with eventual biochemical and symptomatic remission achieved over the course of five months. A fine needle aspiration cytology (FNAC) biopsy was done on the left thyroid that showed a benign follicular nodule. She was followed with serial thyroid USs that demonstrated a slow increase in the sizes of the previous suspicious nodules. A repeat thyroid scan showed elevated uptake with hyperfunctioning nodules in the left-upper and right-lower lobes (38%, 24-hour uptake).

**Table 1:** A comparative assessment of the patient's initial and most recent lab profiles. TSH: thyroid-stimulating hormone; T4: thyroxine; IgG: immunoglobulin G; PTH: parathyroid hormone; Tg-RIA: thyroglobulin radioimmunoassay

Parameter	Latest reference range and unit	Most recent lab	Initial lab
TSH	0.465 - 4.680 uIU/mL	0.758	<0.015
Free T4	0.78 - 2.19 ng/dL	1.88	2.5 (H)
Thyroid-stimulating immunoglobulin	0-130 %	-	369 (H)
Thyroid peroxidase antibodies	<30 IU/mL	-	567 (H)
Triiodothyronine free	2.0 - 4.4 pg/mL	2.2	-
Thyroglobulin antibody	0.0 - 0.9 IU/mL	61.0 (H)	-
Anti-thyroglobulin antibodies	0-40 IU/mL	48 (H)	-
IgG subclass 4	2 - 96 mg/dL	137 (H)	-
PTH, intact	15 - 65 pg/mL	70 (H)	-
Tg-RIA	1.40-29.2 ng/mL	14	-

A repeat fine needle aspiration (FNA) biopsy (FNAB) was done that showed papillary thyroid carcinoma (Bethesda VI) of the left thyroid lobe. She underwent a total thyroidectomy, and pathology reported invasive papillary carcinoma in both the left and right lobes with one metastatic lymph node and carcinoma extending into the adjacent soft tissue (pT4a pretracheal tumor). Subsequently, our patient had a whole body I-131 scan that showed possible thyroid remnants versus local regional metastasis in the neck, as well as possible lung metastasis. A limited evaluation with a non-contrast computed tomography (CT) scan did not show any gross evidence of metastasis. She received a treatment dose of 101.2 mCi of radioactive I-131 with a subsequent significant decrease in thyroglobulin antibody titers from 130 to 14 IU/mL and thyroglobulin <0.1 ng/mL. She is being managed with replacement levothyroxine to target TSH levels between 0.1 and 0.5 and has been doing well.

## Discussion

Thyroid nodules affect 4%-7% of the iodine-sufficient populations, and even more of the iodine-deficient populations, making them one of the most common endocrine problems [4]. Thyroid nodules can be non-functioning (don’t produce hormones) or functioning (produce hormones) [5]. Taking the microscopic and macroscopic factors into consideration, thyroid nodules can be classified as carcinoma, adenoma, or hyperplastic lesions [4]. Thyroid nodules might remain asymptomatic, or they might cause dysphagia, hoarseness, or foreign body sensation [6]. Functioning adenomas are benign with rare incidences of malignancy [7]. Of all the thyroid nodules, 5-10% are considered hyperfunctioning thyroid nodules [8]. Of all the thyroid nodules, 90%-95% are non-functional benign, so they are managed conservatively [4]. TSH level should be measured and scintigraphy should be done if the nodule is functional [9]. On scintigraphy, hot thyroid nodules have increased uptake of the radiotracer compared to the surrounding parenchyma [8]. These hot nodules come in two forms: singular or toxic multinodular [8].

Thyroid carcinomas, which include mainly papillary thyroid carcinoma (PTC) and follicular thyroid carcinoma (FTC), are rare with 1-10 cases for every 100,000 people per year [5]. Age younger than 16 or older than 45 is associated with increased malignancy risk [10]. Papillary and follicular thyroid carcinoma encompass only 1% of all malignancies, but they are the most common endocrine malignancies [4]. The current guidelines suggest the use of FNA-US for nodules with a size of 1 cm or more, or in nodules with suspicious features like microcalcification, Doppler central flow, hypoechogeneity, or border irregularity regardless of the nodule size [10]. FNA has a low diagnostic yield in detecting thyroid malignancy [8]. Due to the increased access to US and FNA cytology, small-sized carcinomas are detected more frequently [10]. A total of 49% of the 53 adult studies demonstrated hot nodules on scintigraphy and carcinoma on pathology [8]. In a study done by Ashcraft and Van Herle, 4% of the hot nodules had malignancy risk [11,12]. Also, in a study done by Dirikoc et al., hot nodules had an 8.5% malignancy risk [13]. It is possible to detect benign nodules and malignant nodules within the same thyroid lobe [8].

Patients with nodular goiter and low TSH have an increased risk of malignancy [14]. A study done by Boelaert et al. that contained 1,500 patients, showed a significant increase in malignancy in patients with high TSH levels compared to those with low TSH levels with an odds ratio of 2.72 [15]. Among the factors that increase the risk of malignancy are male gender, solitary nodule, and younger patients [16]. Fiore et al. showed a decreased prevalence of papillary thyroid carcinoma in patients with low TSH [17]. Hashimoto thyroiditis is associated with a higher malignancy risk than Graves’ disease [18].

FNA is considered the most important method when it comes to thyroid nodules [5]. FNA is used to diagnose and monitor these thyroid nodules [5]. It has a specificity ranging from 72% to 100% and a sensitivity ranging from 65% to 98% [10]. FNA has a 1 to 11% false negative rate and 0 to 7% false positive rate [10]. A study done by Choi et al. showed that 16.1% of FNAB-US images were not reliable, and this was attributed to cystic lesions, macrocalcifications, and physician experience [19].

A study done by Lee et al. showed that thyroid cancer might develop in hyperfunctioning nodules with an estimated risk of 6.5% [20]. In another study, it was shown that hyperfunctioning nodules were malignant in 11% of the cases [21]. The cancer risk of thyroid nodules falls between 5-15%, so this shows that hyperfunctioning thyroid nodules fall within the range of malignant thyroid nodules [2]. This shows that patients with hyperfunctioning thyroid nodules might not be cancer-free [20]. The most common thyroid cancer in hyperfunctioning nodules is follicular thyroid carcinoma followed by papillary thyroid carcinoma [22]. The hyperfunctioning nodules are more likely to metastasize than to stay in the same place [23]. In the study done by Lee et al., there were coexisting thyroid nodules in 87.5% of the cases, but the malignant or hyperfunctioning nodules had a larger size than the other coexisting nodules [20].

The reported annual incidence for patients with Graves’ disease with thyroid cancer is 175/100,000 [24]. The incidence of thyroid cancer in Graves’ disease patients is 2.5% [25]. In a study done by Shapiro et al., the incidence of thyroid cancer in Graves’ disease was 8.7% [26]. This increase is most likely because these patients were diagnosed with cancer based on subtotal thyroidectomy [26]. The aggressiveness of thyroid cancer in Graves’ disease patients is not agreed upon yet; some describe it as more aggressive than in euthyroid patients, while others describe it as similar in aggressiveness [27,28]. Thyroid cancer in Graves’ disease patients is more aggressive than in euthyroid patients and has a poorer clinical outcome with more metastasis and lymph node involvement [29,30]. On the other hand, Hales et al. didn’t find any difference in aggressiveness when they compared thyroid cancer in Graves’ disease patients and euthyroid patients, but the size of the tumor in euthyroid patients was larger than in Graves’ disease patients, which was a major limitation in the study [31]. This shows that smaller tumors in Graves’ disease patients and larger tumors in euthyroid patients have the same prognosis [31]. Studies done by Yano et al. and Edmonds et al. showed that lymph node metastasis, distant metastasis, multifocality, and mortality were not significantly different between thyroid cancer patients with Graves’ disease and euthyroid patients [32,33].

Most carcinomas in Graves’ disease patients were found incidentally upon histological examination postoperatively [34]. A total of 88% of the incidental thyroid cancer in Graves’ disease patients were less than 10 mm in size [35]. Equal size tumors were compared between Graves’ disease and euthyroid patients; patients with Graves’ disease were found to have longer survival and excellent prognosis [36]. Pellegriti et al. did a study with 450 Graves’ disease patients [37]. They found that clinically important thyroid cancer in Graves’ disease patients had more lymph node metastasis, distant metastasis, and even death when compared to incidental cancer in Graves’ disease patients [37]. Lee et al. concluded that the postoperative concentration of thyroglobulin, the levels of the thyroid hormones before giving the antithyroid medications, and the length of thyrotoxic symptoms were not significantly different between silent and clinical thyroid cancer in Graves’ disease patients [38].

The thyroid nodules that are present in Graves’ disease patients are associated with a higher risk of malignancy [3]. A study done by Kraimps et al., which included 557 Graves’ disease patients, showed that 3.8% of the patients had thyroid carcinoma post-thyroidectomy [39]. Pacini et al. found that 22.2% of the patients who had a thyroid nodule with toxic multinodular goiter developed thyroid carcinoma while only 2.9% of the patients who had toxic multinodular goiter with a thyroid nodule developed thyroid carcinoma [40]. This showed the importance of screening the thyroid nodules in Graves’ disease patients [40]. The antithyroid medications used to treat Graves’ disease make cytomorphological changes, which makes it difficult for FNA to differentiate between benign and papillary thyroid carcinomas [3]. The radioactive iodine might cause atypia, which might be mistaken for a thyroid malignancy [41].

According to Ozaki et al., thyroid carcinoma was only found in 0.17% of Graves’ disease patients undergoing radioactive iodine therapy, while it was 2.5% in patients undergoing surgery [42]. This could be because patients who had surgery were more likely to be diagnosed than patients who only got radioactive iodine for treatment [3]. The pathogenesis of Graves’ disease is done by thyroid-stimulating hormone receptor antibody (TSHRAb) stimulating the TSH receptors and activating the downstream activities [3]. This allows the production of thyroid hormones while suppressing the hypothalamus-pituitary-thyroid axis [3]. The prognosis of thyroid cancer is affected by chronic TSH receptor stimulation, so this might explain why Graves’ disease is associated with more aggressive thyroid cancer [3]. 

Since patients with Graves’ disease have more aggressive thyroid cancer, it is recommended to do near-total or total thyroidectomy with central neck dissection [37]. This will treat thyroid cancer, which is more important than treating hyperthyroidism [3]. Subtotal thyroidectomy could be used in the treatment of thyroid cancer in Graves’ disease patients who have a cancer size of less than 10 mm, and it is associated with an excellent prognosis [43]. According to the European and American Thyroid Association, total or near-total thyroidectomy is the gold standard treatment in papillary thyroid microcarcinoma (PTM) when diagnosed preoperatively [3]. If the PTM is found after the surgery in Graves’ disease patients and the PTM is well-differentiated, unifocal, without lymph node metastasis, and without extra thyroid invasion, then no further treatment is required [44].

## Conclusions

Hyperfunctioning nodules are considered benign nodules; on the other hand, cold nodules are considered malignant. The next step after history and physical exam is obtaining serum TSH and US of the thyroid gland. After detecting low TSH, thyroid scintigraphy should be obtained. FNA is done on hypofunctioning nodules that are detected on thyroid scintigraphy. In this paper, we demonstrated that hot nodules with elevated TSH could still be associated with papillary thyroid carcinoma. This raises the question of whether hot nodules are misdiagnosed as benign. Further workup for hot nodules needs to be done.
